# Miliary Tuberculosis with Diffuse Pulmonary and Extrapulmonary Involvement

**DOI:** 10.5334/jbsr.3440

**Published:** 2024-01-27

**Authors:** Hafsa Selmani, Adelard I. De Backer, Bart Ilsen

**Affiliations:** 1 Department of Radiology, University Hospital Brussels, Brussels, Belgium; 2 Department of Radiology, University Hospital Brussels, Brussels, Belgium; 3 Department of Radiology, University Hospital Brussels, Brussels, Belgium

**Keywords:** Miliary tuberculosis, CT, extrapulmonary tuberculosis, random distribution

## Abstract

*Teaching Point:* In patients coming from countries with a high prevalence of tuberculosis and presenting with chronic infectious disease, tuberculosis with pulmonary and/or extrapulmonary involvement should be included in the differential diagnosis.

## Case History

A 48-year-old female originally from Sub-Saharan Africa and residing in Europe for the past 2 years was referred to the emergency department due to fever for several months, increasing fatigue with marked weakness, and abdominal pain. The patient reported weight loss over the preceding months, night sweats, and intermittent episodes of melena, but no respiratory complaints. Laboratory analysis revealed significant anemia, hyponatremia, and evidence of an escalated inflammatory response. Physical examination showed diffuse abdominal tenderness, most pronounced in the lower quadrants without rebound.

**Figure 1:**
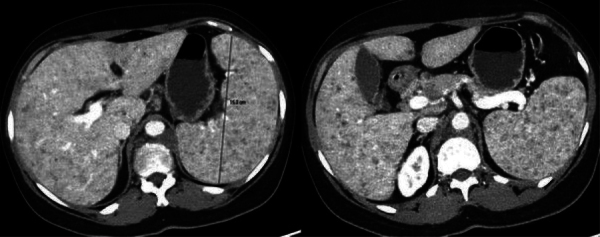
Axial contrast-enhanced CT

**Figure 2:**
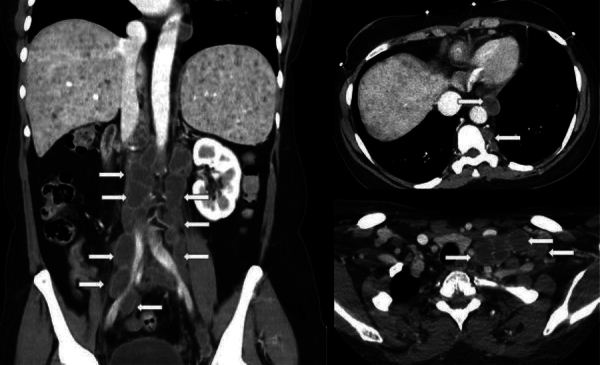
Coronal and axial contrast-enhanced CT

**Figure 3:**
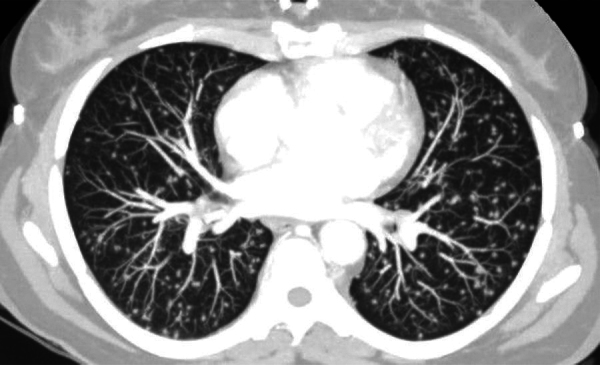
Computed tomography parenchymal window of the lung

Contrast-enhanced computed tomography (CT) showed a significant splenomegaly, measuring up to 15 cm in the axial plane, without evidence of hepatomegaly. The liver and spleen parenchyma displayed a diffuse heterogeneous pattern caused by ill-defined small hypodense nodules measuring up to 5 mm (Fig. 1). A CT scan also highlighted widespread intra-abdominal lymphadenopathies, characterized by peripheral enhancement and central hypodensity, particularly concentrated along the retroperitoneal vascular axes. Adenopathies were also noted in the paravertebral region of the thoracic spine and in the cervical regions (Fig. 2). Additionally, the parenchymal window of the lung bases showed micronodules diffusely spread at random distribution (Fig. 3). A diagnosis of miliary tuberculosis (MT) with involvement of both the lungs, liver, spleen, and lymph nodes was suggested. Subsequently, bronchoalveolar lavage testing and sputum evaluation revealed, respectively, a positive polymerase chain reaction (PCR) test and a low positive result for *Mycobacterium tuberculosis*.

## Comments

MT has been reported to represent approximately 1% to 2% of all tuberculosis cases and 8% of extrapulmonary tuberculosis cases. The resurgence of MT has been particularly noted among immigrants from countries with high tuberculosis prevalence and immunodeficient patients.

MT may occur as a primary infection or can develop months to years after the initial infection, also called a post-primary infection. The characteristic uniform, diffuse, and “at random” distribution of micronodular miliary lesions in the parenchyma of the lungs, liver, or spleen results from lymphohematogenous dissemination of tuberculous infection from a primary site to other organs [1]. In the lungs, MT manifests as interstitial micronodular distributions predominantly present in the lower lobes without bronchial wall thickening. In solid viscera, however, these small nodular lesions may vary in size and in peripheral enhancement due to granulation tissue with central caseation or liquefactive necrosis. An associated splenomegaly is usually noted.

Tuberculous lymphadenopathy is the most common manifestation of abdominal tuberculosis. In MT with abdominal involvement of solid viscera (e.g., liver and spleen), the gastrointestinal tract and peritoneum are usually affected as well. CT findings of tuberculous lymphadenopathy include, as seen in our patient, circular or ovoid lesions showing peripheral and variable enhancement with central low-density.

Antituberculous drugs are generally administered for at least 12 months to treat patients affected by MT.
